# Stretchable nanofibers of polyvinylidenefluoride (PVDF)/thermoplastic polyurethane (TPU) nanocomposite to support piezoelectric response via mechanical elasticity

**DOI:** 10.1038/s41598-022-11465-5

**Published:** 2022-05-18

**Authors:** Nader Shehata, Remya Nair, Rabab Boualayan, Ishac Kandas, Abdulrzak Masrani, Eman Elnabawy, Nada Omran, Mohammed Gamal, Ahmed H. Hassanin

**Affiliations:** 1 Kuwait College of Science and Technology (KCST), 13133 Doha, Kuwait; 2 Center of Smart Materials, Nanotechnology and Photonics (CSMNP), Smart CI Research Center, Alexandria University, Alexandria, 21544 Egypt; 3 Department of Engineering Mathematics and Physics, Faculty of Engineering, Alexandria University, Alexandria, 21544 Egypt; 4 USTAR Bioinnovations Center, Faculty of Science, Utah State University, Logan, UT 84341 USA; 5 Department of Mechanical Engineering, Roberts Engineering Building, University College London (UCL), London, WC1E 7JW UK; 6 Micro System Design and Manufacturing Center, Department of Mechanical Engineering, Bilkent University, Ankara, 06800 Turkey; 7 Material Science and Engineering Department, School of Innovative Design Engineering, Egypt-Japan University of Science and Technology (E-JUST), New Borg El-Arab City, Alexandria, Egypt; 8 Department of Textile Engineering, Faculty of Engineering, Alexandria University, Alexandria, 21544 Egypt

**Keywords:** Materials science, Nanoscience and technology

## Abstract

Interest in piezoelectric nanocomposites has been vastly growing in the energy harvesting field. They are applied in wearable electronics, mechanical actuators, and electromechanical membranes. In this research work, nanocomposite membranes of different blend ratios from PVDF and TPU have been synthesized. The PVDF is responsible for piezoelectric performance where it is one of the promising polymeric organic materials containing β-sheets, to convert applied mechanical stress into electric voltage. In addition, the TPU is widely used in the plastic industry due to its superior elasticity. Our work investigates the piezoresponse analysis for different blending ratios of PVDF/TPU. It has been found that TPU blending ratios of 15–17.5% give higher output voltage at different stresses conditions along with higher piezosensitivity. Then, TPU addition with its superior mechanical elasticity can partially compensate PVDF to enhance the piezoelectric response of the PVDF/TPU nanocomposite mats. This work can help reducing the amount of added PVDF in piezoelectric membranes with enhanced piezo sensitivity and mechanical elasticity.

## Introduction

Over the last few decades, extensive research regarding the use of alternative energy sources has been carried out^1^. This has mainly followed the use of various clean and renewable energy sources due to their sustainability and environmental friendliness^2^. Moreover, energy harvesting technologies have recently been the main focus, where wasted energy from ambient environments is utilized. Such technologies can transform vibrations, heat, light, radiation, wind, and water into electrical energies for low-power devices^1^. Research has also extended further to include energy harvesting in biomedical applications^3^ and offered promising biomedical sensors and wearable electronics^4,5^, due to the ability to harvest kinetic energy in the form of vibrations from direct human activities such as walking, running, and finger tapping to heartbeat and respiration^6,7^. The kinetic energy is harvested based on three transduction mechanisms; piezoelectric, electromagnetic, or electrostatic. Because of their high energy density, simple design, and the ability to be scaled down to micro-and nanoscale devices, piezoelectric energy harvesters have gained the most attention^8–10^. Piezoelectric materials also possess the unique ability to convert mechanical energy into electricity directly, without an external input^11,12^. Therefore, numerous efforts have been put into developing high-performance piezoelectric nanogenerators using organic and inorganic materials^13–15^.

It has been found that organic piezoelectric materials have greater benefits than inorganic materials, including a higher level of processability^16^. Such materials have been seen to be applicable in a wide range of devices, with polymer-based materials being more preferred due to their intrinsic flexible nature, providing a great degree of bending and biodegradability^17,18^. Among all piezoelectric polymers, poly(vinylidene fluoride) (PVDF) films have shown the highest piezoelectric performances to date^19–21^. Due to the polar crystalline nature of PVDF, its ability to produce large voltages with low forces has made it favorable for piezoelectric applications^20,22,23^. The piezoelectric property of PVDF mainly depends on its β-phase, one of its four crystalline phases^22,24^. In addition to its lightweight, flexibility, resistance to solvents, and stability under high electric fields, it is considered as the optimum biomaterial for applications in energy harvesters, force sensors, and transducers.

PVDF nanofibers are the core candidate for such applications, especially wearable and implantable devices. The main techniques used to fabricate such fibers include electrospinning, melt spinning, and centrifugal spinning^25,26^. Electrospinning has been the most promising as it can form nanofibers from solutions or melts with variable diameters. Additionally, it has been reported that the β-phase content in PVDF nanofibers produced by electrospinning is higher than that of PVDF cast films, thereby improving its piezoelectric properties^27^.

Electrospinning also provides the ability to further enhance the piezoelectric properties of the fabricated PVDF nanofibers due to its ability to produce aligned fibers with hollow structures or various additives for improved performance^28^. Such additives include carbon nanotubes (CNTs), graphene, and ZnO^29^. A previous study has successfully produced a nanogenerator from PVDF-ZnO nanocomposite, showing that adding ZnO particles increased its output voltage^30^. Furthermore, piezoelectric nanogenerators incorporating ZnO have successfully been implanted in live rats to harvest energy from heartbeats and breathing motion. That study has shown great potential for using PVDF-ZnO nanogenerators as a power source for implantable biomedical electronic devices, which suggests a strong potential for such devices in applications related to the normalization of the heartbeat and brain stimulation for the treatment of movement disorders^31^. Furthermore, PVDF and its copolymers have been utilized for pressure sensing applications, serving as healthcare monitoring devices for respiration signals. Composites of PVDF and graphene oxides were also developed for multiple sensory applications, exhibiting a high sensitivity for monitoring simultaneous artery pulse pressures and temperatures^32^. PVDF nanofibers have also been explored for the application of blood pressure sensors due to their excellent flexibility^33^, and has successfully been tested on an in vitro model where PVDF thin films were wrapped around the aorta, and periodic signals of output current and voltage were generated with the movement of the artery, showing a great sensitivity.

Compared to multiple composites, the addition of thermoplastic polyurethane (TPU) has shown great potential for enhancing mechanical properties^34^. Such characteristics may be required for applications in wound healing and filtration.

Several studies have investigated PVDF/TPU composite mat characteristics and performance for different electrical and biomedical applications^35–38^. PVDF/TPU electrospun scaffolds were introduced for wound healing, where cell migration and fibroblast activities were seen to be improved due to the piezoelectricity of the composite material^39^. Another study has evaluated the changes in piezoelectric and mechanical properties regarding the addition of TPU with PVDF nanofibers^40^. The results showed more flexibility in dipole excitations inside PVDF due to the elastic content of TPU. (GO)/Bi2S3-PVDF/TPU composite nanofiber mat was developed for photothermal applications^41^, combining GO/Bi2S3 nanoparticles as a photothermal conversion material and electrospun PVDF/TPU membrane as a substrate. The results observed that the hybrid novel mat has about a 95% light absorption rate at a wavelength range of 400–2500 nm. In addition, the presence of TPU significantly improved the mechanical strength of the composite film. TPU and bismuth sodium titanate polycrystalline oxide (Bi_0.5_Na_0.5_TiO_3_; BNT) were blended with (PVDF) and casted with the aid of a blade coater to investigate their effect on the piezoelectric response of composite membranes^42^. The remarkable enhancement in face shear piezoelectric coefficient (d_36_) was revealed by adjusting the blending content of TPU due to micro-pore structure formation, which facilitates charge transfer under different stress types.

In our work, we analyze the detailed mechanical and piezoelectric characteristics of different blending ratios of PVDF and TPU nanofiber mats synthesized by the electrospinning process. In detail, we check the optimum blending ratios to generate the maximum voltage at different applied forces. In addition, we show the impact of frequency of the applied force on the piezoresponse behavior of different blended nanofiber mats. This work is helpful for wearable electronics and energy harvesting units.

## Experimental work

### Materials

Polyvinylidene fluoride (PVDF) (Kynar, Arkema, PA, USA) is supplied by ARKEMA and thermoplastic polyurethane (TPU) with Polydispersity Index (PDI) of 1.83 and molecular weight of 107,020 g mol^−1^ is supplied by (BASF Co., Ltd., Berlin, Germany). Known polymer concentrations have been dispersed in dimethylformamide (DMF 98%, Sigma Aldrich, Taufkirchen, Germany).

### Membrane fabrication

Different blending ratios of PVDF and TPU solution with a constant polymer concentration of 10% were prepared and processed through the electrospinning setup. Comparative study of the effect of TPU addition on the piezoelectric and mechanical properties of PVDF mat has been introduced through five different blending ratios of PVDF/TPU (95:5, 90:10, 87.7:12.5, 85:15, 82.5:17.5, 80:20, 75:25, and 70:30). The electrospinning process was performed by adding 10 mL of polymer solution into a plastic syringe tipped with a stainless steel needle. The positive voltages were provided from a high voltage power supply CZE1000R (Spellman, Hauppauge, NY, USA) to the metallic needle with gauge 18, for application of voltages around 25 kV with a constant feed rate of (1 mL/h) using a NE1000 syringe pump (New Era Pump Systems, Suffolk County, NY, USA). Needle-to-collector distance adjusted to 10 cm. Random PVDF/TPU nanofibers composite was collected on a drum collector covered with aluminum foil and connected to the ground.

### Morphological and physical characterization

The morphology of PVDF/TPU nanofibers (NFs) was observed by scanning electron microscope (JEOL JSM-6010LV-SEM, Tokyo, Japan) with an accelerating voltage of 15 kV. The nanofiber mats were placed on carbon tape fixed on aluminum stubs and sputter-coated with platinum. The diameter of NFs was analyzed using Image-J software (Madison, WI, USA). The average fiber diameter distribution was manually detected by measuring the length through fiber boundaries at different imaging scales (50 µm, 10 µm, and 1 µm). Fourier transform infrared spectrometer (FT-IR) (Vertex 70 FT-IR, Bruker, Billerica, MA, USA) was adjusted in ATR mode. Samples were scanned 120 times at a resolution of 5 cm^−1^ over a range of 4000–400 cm^−1^ to study the chemical functional groups of blended mats.

### Mechanical characterization

Testing the effect of TPU addition on the mechanical properties of the fabricated mats was performed by cutting the nanofiber membranes into equal rectangular pieces (1 × 6 cm). The samples were fixed between holding frames with a gauge length of 4 cm. A universal testing machine (TENSO LAB 5000, Mesdan, Italy) was used to perform the stress–strain curve. The tensile test was conducted at a strain rate equal to 10 mm/min with zero initial loads using a load cell of 100 N.

### Piezoelectric characterization

**Figure 1:**
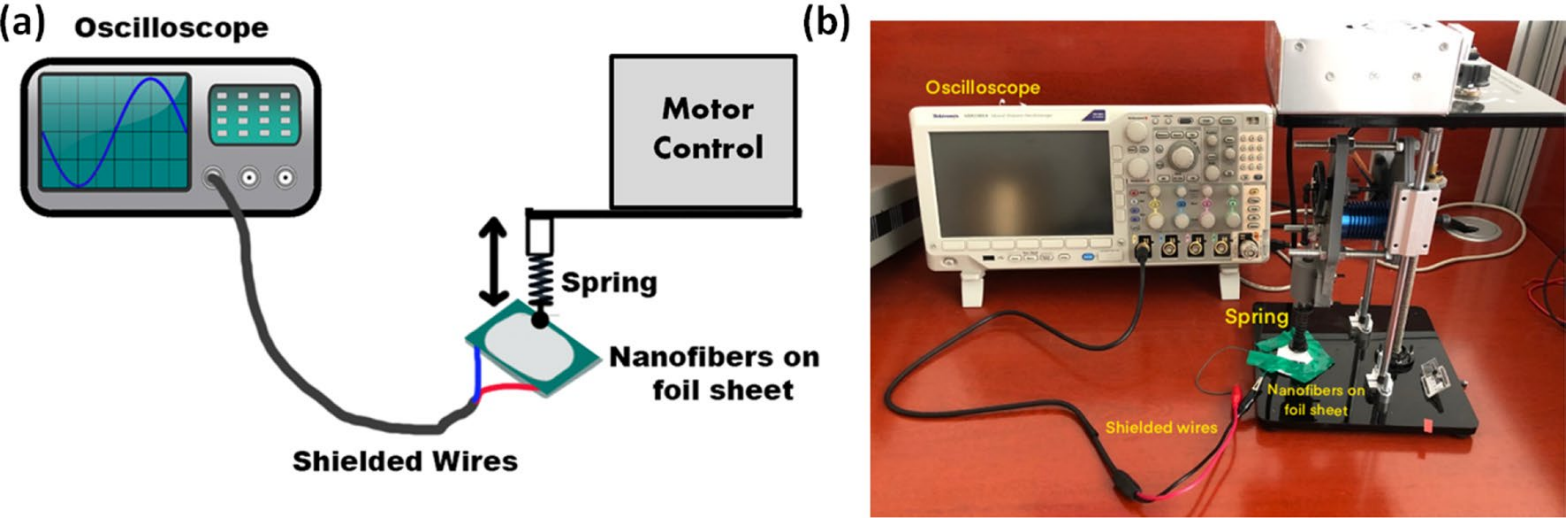
Schematic representation of the piezoelectric characterization set-up (**a**), and a picture of real set-up (**b**).

The synthesized PVDF/TPU nanofiber membranes were tested under a cyclic load using an excitation instrument constructed for this purpose (Fig. 1). The instrument consists of a lightweight spring plunger assembly that oscillates vertically. The excitation frequency is controlled by varying the speed of the brushless DC motor driving the plunger using an electronic speed controller. The sample is sandwiched between two sheets of foil connected through shielded wires to a high impedance oscilloscope (Tektronix MDO3014) and then placed underneath the plunger. The maximum load is controlled by controlling the height of the plunger and therefore changing the compression distance of the spring upon engagement with the sample during operation. The maximum applied force ranged between 1 and 3 N, and peak-to-peak voltage was measured accordingly.

### Impulse loading characterisation

**Figure 2:**
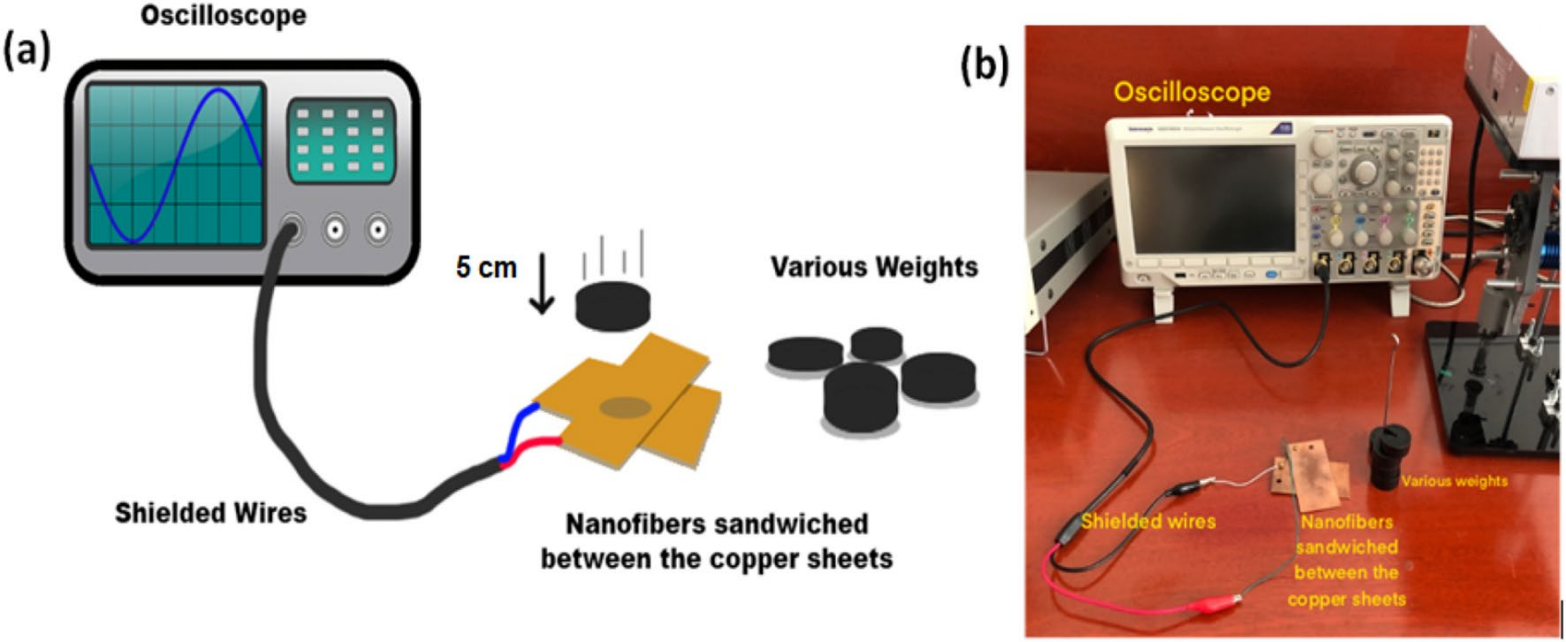
Schematic representation of the impulse loading set-up (**a**), and a picture of real set-up (**b**).

Piezoelectric voltage signals from the PVDF/TPU nanofibers were analyzed through a simple impulse loading setup, as shown in Figure 2. The nanofiber mats were placed between two copper sheets, connected to a high impedance oscilloscope through shielded wires, and exposed to different weights for the impulse loading test. The weights, ranging from 50 to 250 g, were dropped onto the sandwiched nanofibers from a fixed height of 5 cm. The resulting voltage was then detected and assessed (Fig. 2).

## Results and discussions

### Morphological characterization

**Figure 3:**
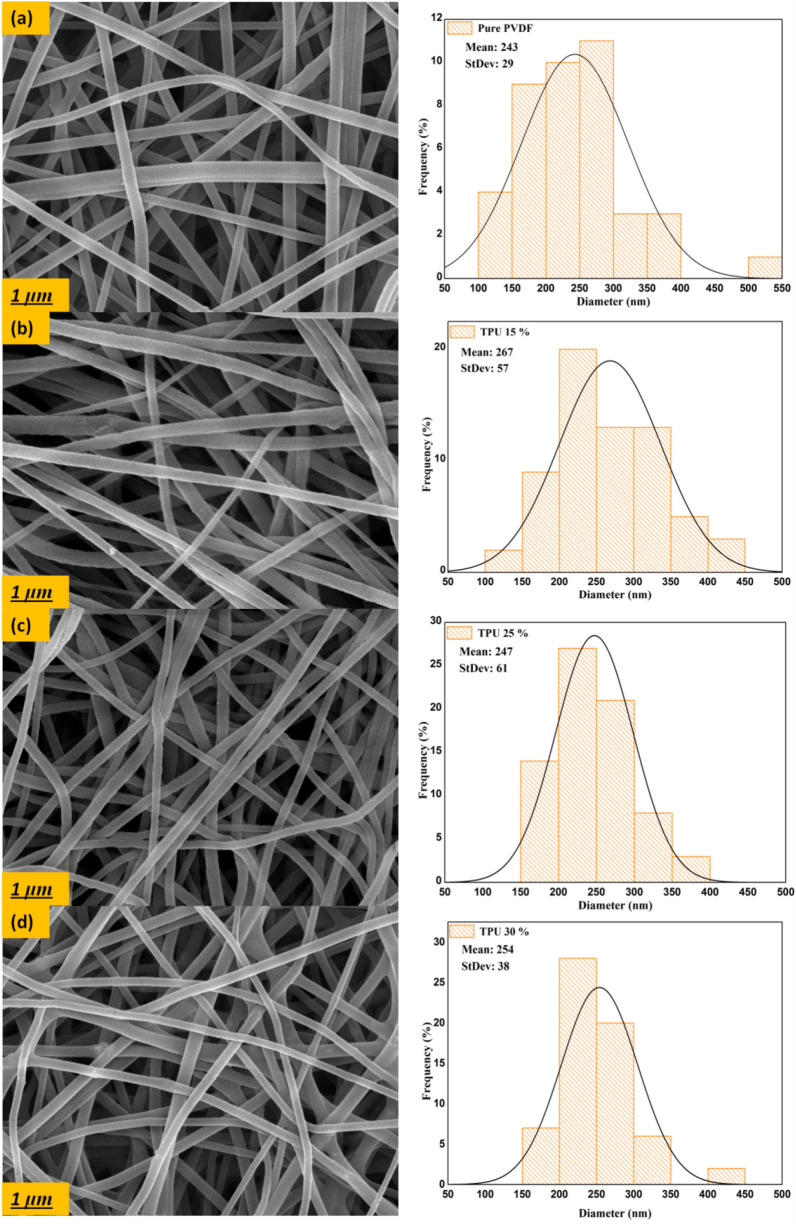
SEM images of pure PVDF (**a**), TPU 15% (**b**), TPU 25% (**c**), and TPU 30% (**d**) composite nanofibers membranes.

Figure 3 shows the SEM images of PVDF/TPU composite nanofibers. The images show homogenous fibers distribution with minimum beads formation. The average fiber diameter was calculated, and a histogram of fiber distribution was presented in Fig. 3. The results show an average fibers diameter for pure PVDF and blended composite mats in the range of 254 nm to 267 nm. It was observed that the TPU addition didn’t significantly affect the fiber diameter, which ensures the high compatibility and homogeneity of the mixed polymer solution.

### Physical characterization

**Figure 4:**
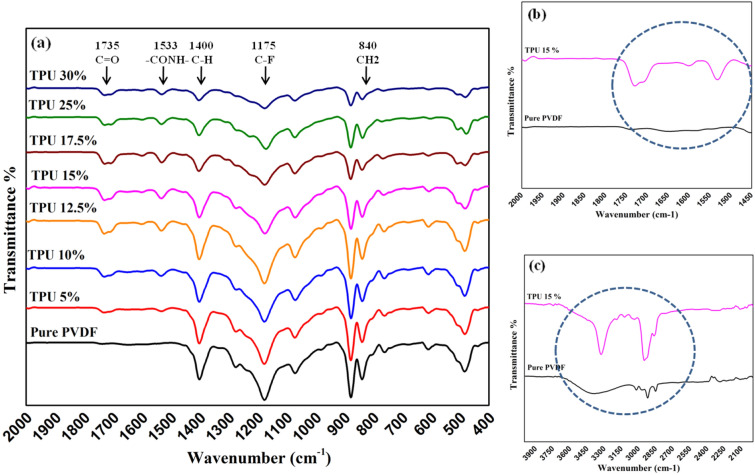
FT-IR curves for PVDF/TPU composite nanofibers.

The FT-IR spectra of nanofibrous composite membranes are shown in Fig. 4. The FT-IR data is introduced to identify the crystalline phases of PVDF. PVDF can be formed in different five crystalline phases (α, β, γ, δ, and ε). The α-phase is considered the most obtained and stable non-polar phase of PVDF, while the β-phase is responsible for enhancing the piezoelectric properties. It was found that the electrospinning process can improve the piezoelectricity of PVDF by increasing the β-phase content; this can be attributed to the effect of the high electric field, which induces the dipoles to be aligned in the same direction, normal to the chain axis forming spontaneous polarization and exhibiting a strong piezoelectric effect. As shown in Fig. 4a, the graph shows the main characteristic bands for PVDF at 840 cm^−1^ for CH_2_ rocking, C–C and CF_2_ stretching, 1175 and 1400 cm^−1^ for C-F, and C-H vibrations, respectively^43–45^. While the characteristic bands of TPU appeared at 1533, 1735, 2971, 3365 cm^−1^ corresponding to –CONH– asymmetrical bond, C=O, C–H, and N–H stretching, respectively^46,47^. The resultant data in Fig. 4a clarifies the intensity decrease in PVDF absorption bands with increasing TPU content. Moreover, the characteristic peaks of TPU strongly appeared with higher TPU concentrations addition compared to the pure PVDF, as presented in Fig. 4b,c.

The beta-phase content was calculated according to the following equation derived from Beer-Lambert law;

1 $$F\left(\beta \right)=\frac{{A}_{\beta }}{1.26 {A}_{\alpha }+{A}_{\beta }}$$

where A_α_ and A_β_ are the intensities of absorbance bands at 764 cm^−1^ and 840 cm^−1^ respectively.

As shown in Table 1, remarkably high polar beta-sheets content was observed for pure PVDF and TPU 15%, confirming the superior piezoelectric response of the TPU 15% composite mat. It was reported that the improvement in β-phase content in electrospun nanofibers is attributed to polymer jet stretching under a high electric field inside the electrospinning process^45^. Hence, the high β-phase content for TPU 15% can be attributed to the effect of TPU mechanical elasticity on facilitating the reorientation of electric dipoles inside the composite nanofiber under the exposure of applied mechanical excitation^42^. However, the more increase of TPU content beyond 15%; the composite loses the resulting polarizability and the corresponding beta-sheets content from the PVDF.

**Table 1:** Calculated fraction of β-phase content for PVDF/TPU composite nanofibers.

Sample	A (α)	A (β)	F (β) content (%)
Pure PVDF	0.029	0.138	79.06
TPU 5%	0.034	0.147	77.43
TPU 10%	0.039	0.132	72.87
TPU 12.5%	0.042	0.157	74.79
TPU 15%	0.015	0.076	80.00
TPU 17.5%	0.022	0.065	70.10
TPU 25%	0.030	0.117	75.58
TPU 30%	0.011	0.041	74.73

### Mechanical analysis

**Figure 5:**
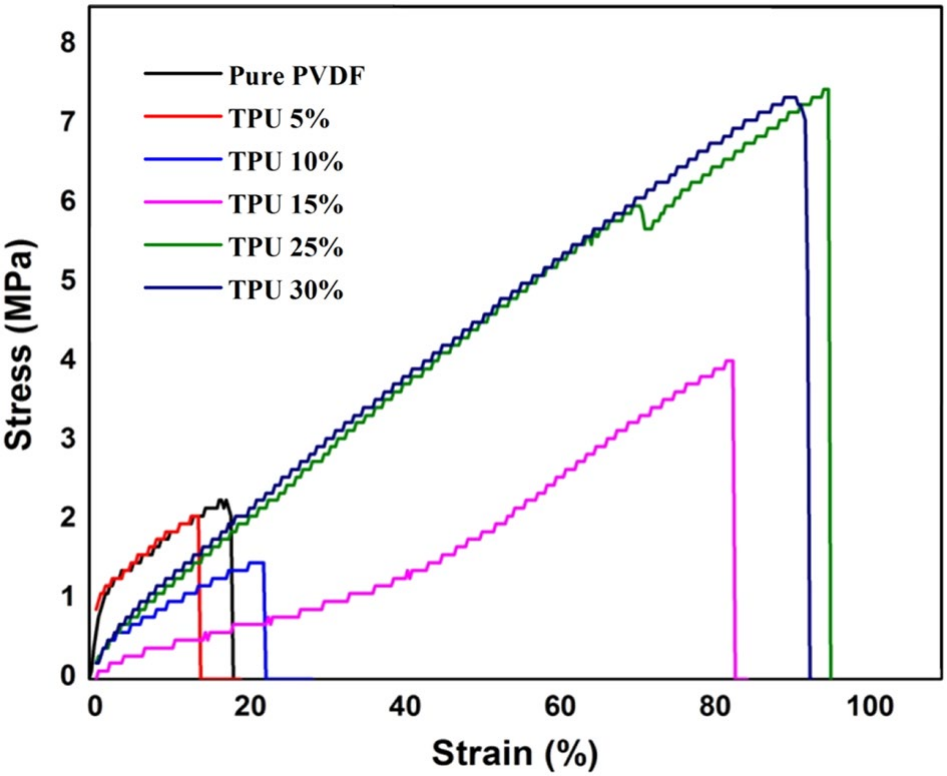
Stress–strain curves of PVDF/TPU composite nanofibers.

Figure 5 shows the stress–strain curves of PVDF/TPU composite nanofiber membranes. It was clearly observed that the addition of TPU significantly improved the mechanical properties of the produced membranes. TPU 25% and 30% revealed nearly similar maximum tensile strength of ~ 7 MPa and breaking strain of ~ 97%. While the pure PVDF and low TPU concentrations (5% and 10%) showed low elasticity with tensile strength below 2 MPa, and elongation at breakage of 23%. As seen, increasing the TPU content up to 30% has increased membrane’s elasticity more than fourfold of the pure PVDF. TPU 15% showed optimum behavior between high and low TPU concentrations with a tensile strength of 3.8 MPa and a breaking strain of 82%. These superior mechanical elasticity characteristics of PVDF/TPU composite membranes make them a good candidate for several applications, which need high elasticity, such as strain gauge, wound healing, and air filtration^39,48,49^.

### Piezoelectric characterization

#### Force-voltage analysis of PVDF/TPU composite nanofibers

Regarding the Force-Voltage analysis for different PVDF/TPU concentrations, Table 2 highlights each sample tested along with the resulting voltage at the minimum and maximum forces used. It can be noted that the resulting voltage appeared to increase by increasing the force applied and the TPU concentration. However, it is seen that a great increase in the TPU concentrations resulted in lower peak-to-peak voltages or provided disruptive results. The PVDF/TPU composite nanofiber mats with a concentration of 15% TPU appeared to have the highest value, while the highest concentration of TPU (30%) provided unstable results. This result was confirmed with a recent study that investigates the effect of TPU and bismuth sodium titanate polycrystalline oxide (BNT) addition on the piezoelectric coefficient of PVDF^42^. The experiments showed that the intensity of face shear electro-mechanical coupling is remarkably affected by TPU addition. Significant enhancement of piezoelectric coefficient d_36_ is obtained when a small portion of TPU (≤ 5%) is introduced into the composite. Within the range of 5–20%, a nearly linear increase of d_36_can be achieved; then, it decreases at higher TPU concentration (> 20%)^42^. The polarization inside the nanocomposite is mainly in the direction of mat’s thickness due to the electric field direction inside the electrospinning process. However, TPU mostly affects a shear strain. Based on the resulted improvement of piezoresponse, we think that such shear strain helps in a better orientation of polarizability to make more aligned dipoles in the thickness’ direction, and consequently enhanced generated output voltage at the sample applied normal force.

**Table 2:** Resulting voltage at the maximum and minimum applied force at a rate of 8 Hz.

Sample	Applied frequency of 8 Hz			Applied frequency of 16 Hz		
	V_Output_ at applied force 1 N, (mV)	V_Output_ at applied force 3 N, (mV)	Piezoresponse Sensitivity (mV/N)	V_Output_ at applied force 1 N, (mV)	V_Output_ at applied force 3 N, (mV)	Piezoresponse sensitivity (mV/N)
Pure PVDF	350 ± 52	575 ± 58	112	373 ± 27	610 ± 34	118
PVDF/TPU (5%)	200 ± 50	320 ± 70	60	340 ± 39	400 ± 51	30
PVDF/TPU (10%)	300 ± 42	600 ± 60	150	1400 ± 70	1800 ± 66	200
PVDF/TPU (12.5%)	973 ± 47	1872 ± 55	449	973 ± 49	1138 ± 63	82
PVDF/TPU (15%)	2300 ± 62	3700 ± 55	700	3000 ± 62	3600 ± 80	300
PVDF/TPU (17.5%)	1230 ± 76	3240 ± 89	1005	800 ± 50	3069 ± 72	1134
PVDF/TPU (25%)	200 ± 53	320 ± 48	60	500 ± 61	505 ± 55	2.5
PVDF/TPU (30%)	< 100	200 ± 31	50	200 ± 29	300 ± 33	50

#### Effect of different vibration frequencies on the output voltage

**Figure 6:**
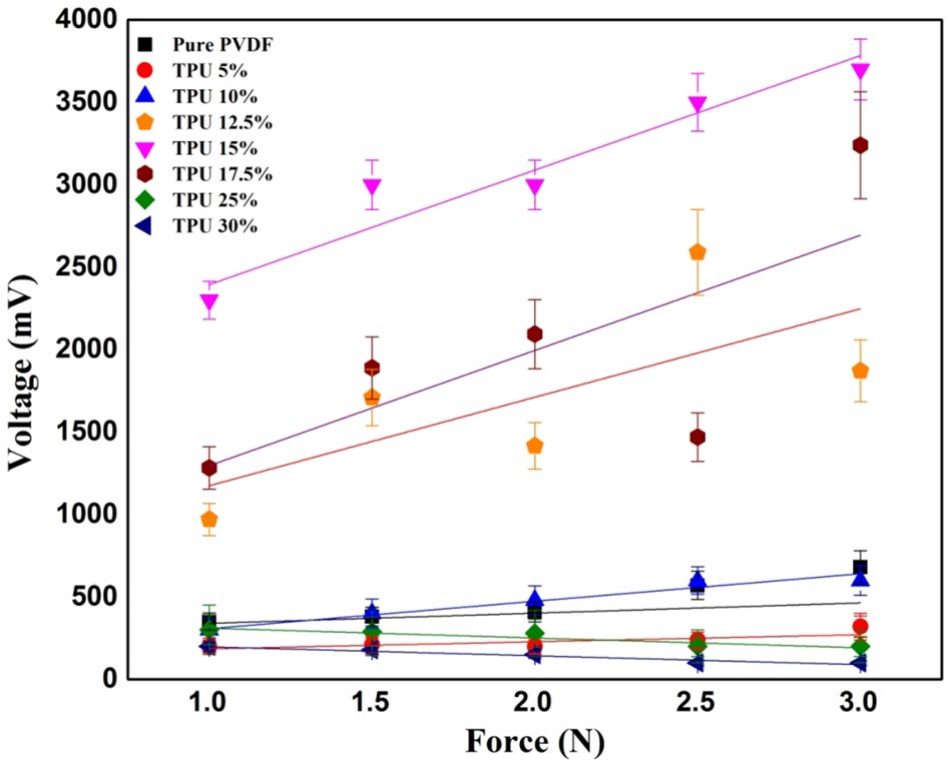
Relation between peak-to-peak voltage and applied forces at a frequency of 8 Hz.

**Figure 7:**
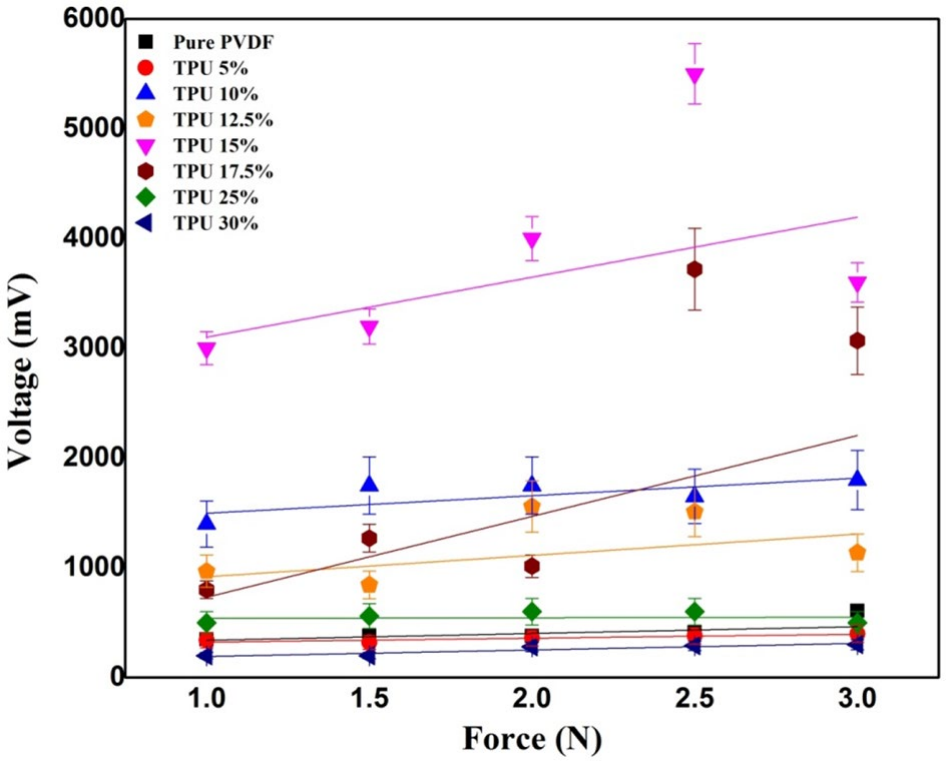
Relation between peak-to-peak voltage and applied forces at a frequency of 16 Hz.

**Figure 8:**
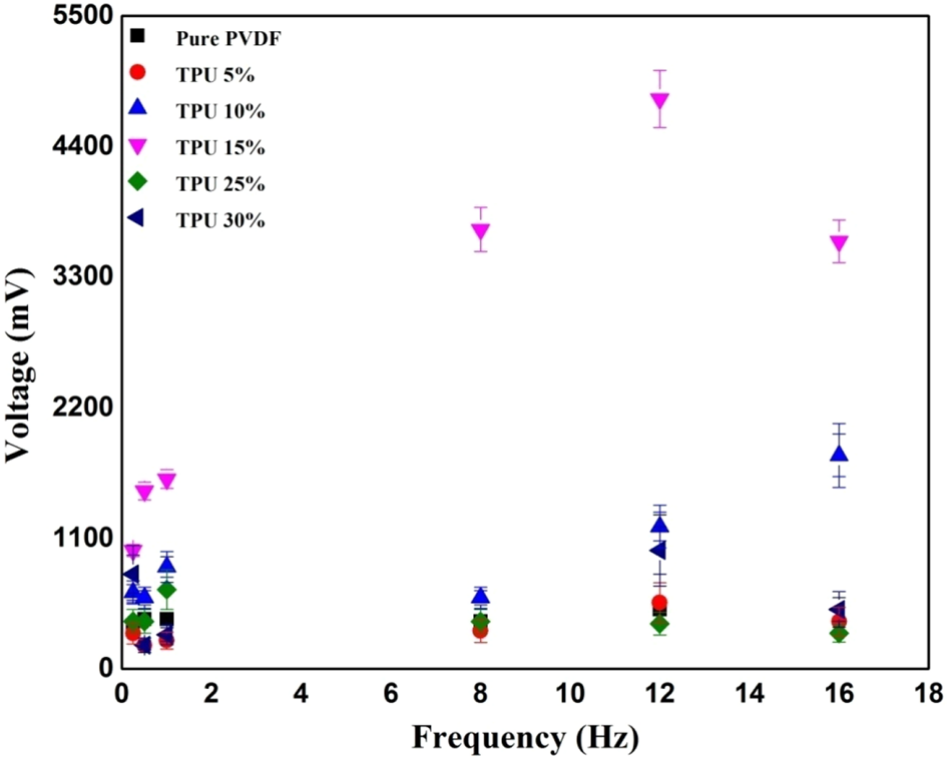
Output voltage at different mechanical frequencies with an applied force of 3 N.

To better highlight the effects of frequency (f) on the different PVDF/TPU composites, a comparison has been made between PVDF/TPU 30% and PVDF/TPU 10% with cyclic forces applied at a rate of 16 Hz and 8 Hz. It can be concluded that relatively lower TPU concentrations responded better to mechanical vibrational frequencies. In comparison, higher TPU concentration in the PVDF/TPU composite nanofibers resulted in unstable peaks and an undetectable electrical potential. Both Figs. 6 and 7 show the relation between peak-to-peak voltage and applied forces on PVDF/TPU composite mats at different constant frequencies of 8 Hz and 16 Hz, respectively. Figure 8 shows that the 15% of TPU is the best blending ratio to generate the maximum output voltage at different applied mechanical vibrational frequencies, along with a better linear behavior within voltage-force relation compared to other around blending ratios, such as 12.5 and 17.5 wt%. However, it can be observed that the 17.5 wt% shows a better piezosensitivity compared to other blending ratios. Therefore, it can be concluded that the range between 15 and 17.5 wt% of TPU blending ratio shows the best piezoelectric performance from the perspective of output voltage and piezosensitivity.

#### Impulse loading test of PVDF/TPU composite nanofibers

**Figure 9:**
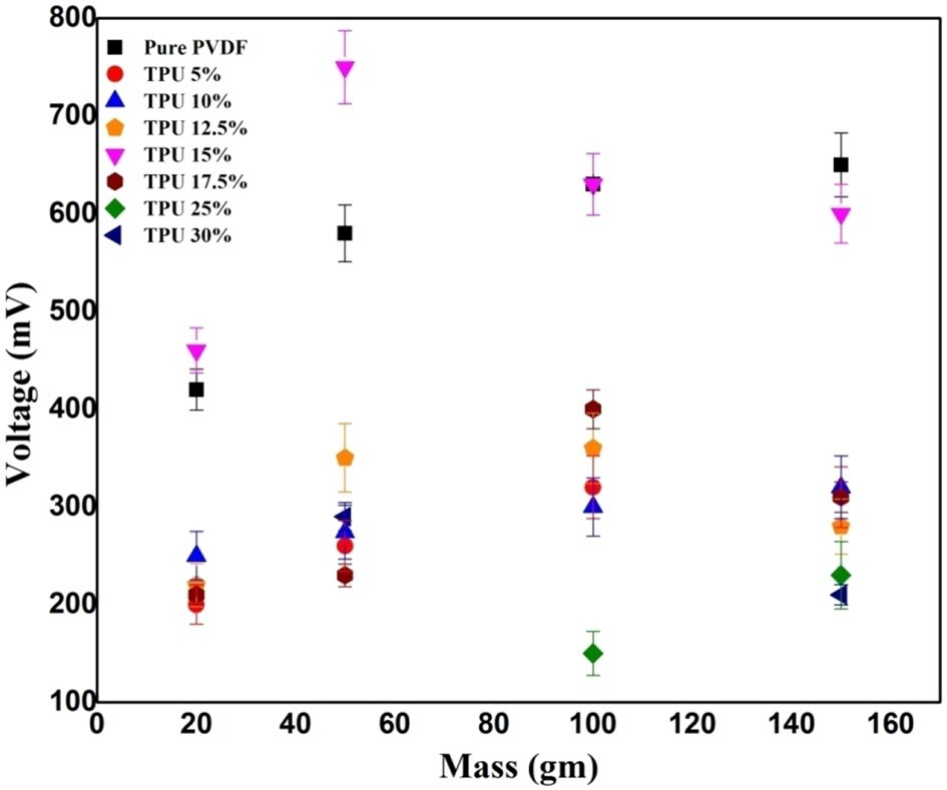
Piezoelectric response of different PVDF/TPU composite nanofiber mats under different impulse weight loading from 5 cm height.

The piezoelectric response of nanofiber mats from different PVDF/TPU composites was analyzed under impulse loading impact from a fixed height of 1 cm. Within most samples, it was observed that the resulting voltage increased while increasing the exposed weight as shown in Fig. 9. Relatively higher output voltage resulted for pure PVDF, while PVDF/TPU blended composite mats showed an apparent decrease in electrical potential at the highest exposed weight (150 gm). In addition, it was observed that TPU 15% had output voltage values relative or similar to pure PVDF, compared to the other samples.

## Conclusion

In this work, we investigated the characteristics of piezoelectric elastic nanocomposites mats. The synthesized nanofiber mats were composed of PVDF with TPU. The piezoelectric performance of the nanocomposite is related to PVDF, and the elasticity feature is related to the blended TPU. Our synthesized mats have been used to generate electric voltage under the effect of different mechanical excitations, such as mechanical stresses, with both controlled forces and vibration frequencies, along with impulse loading via falling masses. The optimum piezoelectric response is found at a blending ratio of TPU between 15 and 17.5 wt%, based on output voltage and piezosensitivity. Although the ratio of PVDF is reduced, the mechanical elasticity of blended TPU causes an improvement in the piezoelectric response of the nanocomposite. This conclusion has been supported by different measurements of piezoelectric voltage at different amplitudes and frequencies of vibrational forces, along with impulsive fallen masses. Meanwhile, FTIR analysis showed that the beta-sheets of 85:15 nanocomposite are nearly equal to the pure PVDF nanofiber mats. This innovative elastic-piezo nanocomposite can be applied within energy harvesting membranes and wearable electronics.

## References

[1] Jurasz J, Canales F, Kies A, Guezgouz M, Beluco A (2020). A review on the complementarity of renewable energy sources: Concept, metrics, application and future research directions. Sol. Energy.

[2] Dyatlov, S., Didenko, N., Ivanova, E., Soshneva, E. & Kulik, S. Prospects for alternative energy sources in global energy sector. In *IOP Conference Series: Earth and Environmental Science *012014 (2020).

[3] Mokhtari F, Azimi B, Salehi M, Hashemikia S, Danti S (2021). Recent advances of polymer-based piezoelectric composites for biomedical applications. J. Mech. Behav. Biomed. Mater..

[4] Shehata N, Hassanin AH, Elnabawy E, Nair R, Bhat SA, Kandas I (2020). Acoustic energy harvesting and sensing via electrospun PVDF nanofiber membrane. Sensors.

[5] Zhou H, Zhang Y, Qiu Y, Wu H, Qin W, Liao Y (2020). Stretchable piezoelectric energy harvesters and self-powered sensors for wearable and implantable devices. Biosens. Bioelectron..

[6] Liu Y, Khanbareh H, Halim MA, Feeney A, Zhang X, Heidari H (2021). Piezoelectric energy harvesting for self-powered wearable upper limb applications. Nano Select.

[7] Zhu M, Yi Z, Yang B, Lee C (2021). Making use of nanoenergy from human–nanogenerator and self-powered sensor enabled sustainable wireless iot sensory systems. Nano Today.

[8] Covaci C, Gontean A (2020). Piezoelectric energy harvesting solutions: A review. Sensors.

[9] Sezer N, Koç M (2020). A comprehensive review on the state-of-the-art of piezoelectric energy harvesting. Nano Energy.

[10] Atif R, Khaliq J, Combrinck M, Hassanin AH, Shehata N, Elnabawy E (2020). Solution blow spinning of polyvinylidene fluoride based fibers for energy harvesting applications: A review. Polymers.

[11] Shivashankar P, Gopalakrishnan S (2020). Review on the use of piezoelectric materials for active vibration, noise, and flow control. Smart Mater. Struct..

[12] Rincón-Quintero, A. et al. Generation and capture of electric energy using piezoelectric materials: a review. In *IOP Conference Series: Materials Science and Engineering*, 012031 (2021).

[13] Sahoo S, Walke P, Nayak SK, Rout CS, Late DJ (2021). Recent developments in self-powered smart chemical sensors for wearable electronics. Nano Res..

[14] Wang F, Sun H, Guo H, Sui H, Wu Q, Liu X (2021). High performance piezoelectric nanogenerator with silver nanowires embedded in polymer matrix for mechanical energy harvesting. Ceram. Int..

[15] Rana MM, Khan AA, Huang G, Mei N, Saritas R, Wen B (2020). Porosity modulated high-performance piezoelectric nanogenerator based on organic/inorganic nanomaterials for self-powered structural health monitoring. ACS Appl. Mater. Interfaces..

[16] Li Q, Zhao J, He B, Hu Z (2021). Solution processable poly (vinylidene fluoride)-based ferroelectric polymers for flexible electronics. APL Mater..

[17] Xu Q, Gao X, Zhao S, Liu YN, Zhang D, Zhou K (2021). Construction of bio-piezoelectric platforms: From structures and synthesis to applications. Adv. Mater..

[18] Keum K, Kim JW, Hong SY, Son JG, Lee SS, Ha JS (2020). Flexible/stretchable supercapacitors with novel functionality for wearable electronics. Adv. Mater..

[19] Cho Y, Jeong J, Choi M, Baek G, Park S, Choi H (2022). BaTiO_3_@ PVDF-TrFE nanocomposites with efficient orientation prepared via phase separation nano-coating method for piezoelectric performance improvement and application to 3D-PENG. Chem. Eng. J..

[20] Sukumaran S, Chatbouri S, Rouxel D, Tisserand E, Thiebaud F, Ben Zineb T (2021). Recent advances in flexible PVDF based piezoelectric polymer devices for energy harvesting applications. J. Intell. Mater. Syst. Struct..

[21] Sharafkhani S, Kokabi M (2021). High performance flexible actuator: PVDF nanofibers incorporated with axially aligned carbon nanotubes. Compos. Part B. Eng..

[22] He Z, Rault F, Lewandowski M, Mohsenzadeh E, Salaün F (2021). Electrospun PVDF nanofibers for piezoelectric applications: A review of the influence of electrospinning parameters on the β phase and crystallinity enhancement. Polymers.

[23] Zhang S, Zhang B, Zhang J, Ren K (2021). Enhanced piezoelectric performance of various electrospun PVDF nanofibers and related self-powered device applications. ACS Appl. Mater. Interfaces..

[24] Ghafari E, Jiang X, Lu N (2018). Surface morphology and beta-phase formation of single polyvinylidene fluoride (PVDF) composite nanofibers. Adv. Compos. Hybrid Mater..

[25] Song J, Kim M, Lee H (2020). Recent Advances on nanofiber fabrications: Unconventional state-of-the-art spinning techniques. Polymers.

[26] Alghoraibi I, Alomari S (2018). Different methods for nanofiber design and fabrication. Handb. nanofibers.

[27] Elnabawy E, Farag M, Soliman A, Mahmoud K, Shehata N, Nair R (2021). Solution blow spinning of piezoelectric nanofiber mat for detecting mechanical and acoustic signals. J. Appl. Polym. Sci..

[28] Gee S, Johnson B, Smith A (2018). Optimizing electrospinning parameters for piezoelectric PVDF nanofiber membranes. J. Membr. Sci..

[29] Shehata N, Elnabawy E, Abdelkader M, Hassanin AH, Salah M, Nair R (2018). Static-aligned piezoelectric poly (vinylidene fluoride) electrospun nanofibers/MWCNT composite membrane: Facile method. Polymers.

[30] Choi M, Murillo G, Hwang S, Kim JW, Jung JH, Chen C-Y (2017). Mechanical and electrical characterization of PVDF-ZnO hybrid structure for application to nanogenerator. Nano Energy.

[31] Mahanty B, Ghosh SK, Jana S, Mallick Z, Sarkar S, Mandal D (2021). ZnO nanoparticle confined stress amplified all-fiber piezoelectric nanogenerator for self-powered healthcare monitoring. Sustain. Energy Fuels.

[32] Park J, Kim M, Lee Y, Lee HS, Ko H (2015). Fingertip skin–inspired microstructured ferroelectric skins discriminate static/dynamic pressure and temperature stimuli. Sci. Adv..

[33] Sharma T, Aroom K, Naik S, Gill B, Zhang JX (2013). Flexible thin-film PVDF-TrFE based pressure sensor for smart catheter applications. Ann. Biomed. Eng..

[34] Aurilia M, Piscitelli F, Sorrentino L, Lavorgna M, Iannace S (2011). Detailed analysis of dynamic mechanical properties of TPU nanocomposite: The role of the interfaces. Eur. Polymer J..

[35] Lin M-F, Xiong J, Wang J, Parida K, Lee PS (2018). Core-shell nanofiber mats for tactile pressure sensor and nanogenerator applications. Nano Energy.

[36] Roy K, Ghosh SK, Sultana A, Garain S, Xie M, Bowen CR (2019). A self-powered wearable pressure sensor and pyroelectric breathing sensor based on GO interfaced PVDF nanofibers. ACS Appl. Nano Mater..

[37] Kweon OY, Lee SJ, Oh JH (2018). Wearable high-performance pressure sensors based on three-dimensional electrospun conductive nanofibers. NPG Asia Mater..

[38] Lee J, Kim S, Lee J, Yang D, Park BC, Ryu S (2014). A stretchable strain sensor based on a metal nanoparticle thin film for human motion detection. Nanoscale.

[39] Guo H-F, Li Z-S, Dong S-W, Chen W-J, Deng L, Wang Y-F (2012). Piezoelectric PU/PVDF electrospun scaffolds for wound healing applications. Colloids Surf., B.

[40] Elnabawy E, Hassanain AH, Shehata N, Popelka A, Nair R, Yousef S (2019). Piezoelastic PVDF/TPU nanofibrous composite membrane: fabrication and characterization. Polymers.

[41] Yang W, Li Y, Feng L, Hou Y, Wang S, Yang B (2020). GO/Bi2S3 doped PVDF/TPU nanofiber membrane with enhanced photothermal performance. Int. J. Mol. Sci..

[42] Dai Z, Feng Z, Feng C, Meng L, Li C, Wang C (2021). Thermoplastic polyurethane elastomer induced shear piezoelectric coefficient enhancement in bismuth sodium titanate–PVDF composite films. J. Appl. Polym. Sci..

[43] Mahato P, Seal A, Garain S, Sen S (2015). Effect of fabrication technique on the crystalline phase and electrical properties of PVDF films. Mater. Sci.-Pol..

[44] Kaspar P, Sobola D, Částková K, Knápek A, Burda D, Orudzhev F (2020). Characterization of polyvinylidene fluoride (PVDF) electrospun fibers doped by carbon flakes. Polymers.

[45] Sengupta D, Kottapalli AGP, Chen SH, Miao JM, Kwok CY, Triantafyllou MS (2017). Characterization of single polyvinylidene fluoride (PVDF) nanofiber for flow sensing applications. AIP Adv..

[46] Gergeroglu H, Huseyin A (2017). Functional composite nanofibers derived from natural extract of Satureja hortensis. Anadolu Univ. J. Sci. Technol. A-Appl. Sci. Eng..

[47] Shaker A, Hassanin AH, Shaalan N, Hassan M, Abd El-Moneim A (2019). Micropatterned flexible strain gauge sensor based on wet electrospun polyurethane/PEDOT: PSS nanofibers. Smart Mater. Struct..

[48] Chen R, Zhang X, Wang P, Xie K, Jian J, Zhang Y (2018). Transparent thermoplastic polyurethane air filters for efficient electrostatic capture of particulate matter pollutants. Nanotechnology.

[49] Tang J, Wu Y, Ma S, Yan T, Pan Z (2022). Flexible strain sensor based on CNT/TPU composite nanofiber yarn for smart sports bandage. Compos. Part B Eng..

