# Author Correction: Stretchable nanofibers of polyvinylidenefluoride (PVDF)/thermoplastic polyurethane (TPU) nanocomposite to support piezoelectric response via mechanical elasticity

**DOI:** 10.1038/s41598-022-15428-8

**Published:** 2022-06-28

**Authors:** Nader Shehata, Remya Nair, Rabab Boualayan, Ishac Kandas, Abdulrzak Masrani, Eman Elnabawy, Nada Omran, Mohammed Gamal, Ahmed H. Hassanin

**Affiliations:** 1 Kuwait College of Science and Technology (KCST), 13133 Doha, Kuwait; 2 Center of Smart Materials, Nanotechnology and Photonics (CSMNP), Smart CI Research Center, Alexandria University, Alexandria, 21544 Egypt; 3 Department of Engineering Mathematics and Physics, Faculty of Engineering, Alexandria University, Alexandria, 21544 Egypt; 4 USTAR Bioinnovations Center, Faculty of Science, Utah State University, Logan, UT 84341 USA; 5 Department of Mechanical Engineering, Roberts Engineering Building, University College London (UCL), London, WC1E 7JW UK; 6 Department of Mechanical Engineering, Micro System Design and Manufacturing Center, Bilkent University, Ankara, 06800 Turkey; 7 Material Science and Engineering Department, School of Innovative Design Engineering, Egypt-Japan University of Science and Technology (E-JUST), New Borg El‑Arab City, Alexandria, Egypt; 8 Department of Textile Engineering, Faculty of Engineering, Alexandria University, Alexandria, 21544 Egypt

Correction to: *Scientific Reports* 10.1038/s41598-022-11465-5, published online 18 May 2022

The original version of this Article contained an error in the spelling of the author Abdulrzak Masrani which was incorrectly given as Abdulrazak Masrani.

The original Article has been corrected.

